# Persistent Unilateral Nerve Paresthesia Following Suspected Viral Infection

**DOI:** 10.1155/crid/3340901

**Published:** 2026-02-24

**Authors:** Nam Nguyen, Willow Meline, Elborz Safarzadeh

**Affiliations:** ^1^ Private Practice, Missouri City, Texas, USA; ^2^ Private Practice, Richmond, Texas, USA; ^3^ Private Practice, Katy, Texas, USA

**Keywords:** facial paresthesia, infraorbital nerve, nerve block, post-COVID-19 neuropathy, trigeminal mononeuropathy

## Abstract

The infraorbital nerve, a terminal branch of the maxillary division (V2) of the trigeminal nerve, supplies sensation to the upper lip, lower eyelid, and midface. Neuropathy in this distribution typically presents as paresthesia with or without anesthesia, dysesthesia, and/or allodynia. While trauma, dental pathology, sinusitis, and certain neurological conditions are possible causes, emerging literature has identified post‐viral neuropathies, including cranial mononeuropathies, as potential sequelae of COVID‐19. This case highlights a rare presentation of presumed post‐viral infraorbital neuropathy following an upper respiratory illness suspected to be COVID‐19. A 34‐year‐old male with controlled hypertension developed unilateral paresthesia of the upper left lip and infraorbital region following a presumed viral illness. Multidisciplinary evaluation included imaging (CT, panoramic, and intraoral radiographs), nasal endoscopy, bloodwork, and dental assessment. An infraorbital nerve block with bupivacaine was used diagnostically to confirm localization. No dental, neoplastic, or significant sinus pathology was identified. Symptoms persisted despite corticosteroids and antibiotics. Infraorbital nerve block reproduced symptoms of paresthesia and temporarily eliminated pain, confirming the affected nerve region. Given the timing postinfection and absence of structural causes, a working diagnosis of post‐viral infraorbital neuropathy was established. This case underscores the importance of including post‐viral neuropathy in the differential diagnosis of facial paresthesia, particularly following respiratory infections like COVID‐19. Diagnostic nerve blocks can aid both localization and symptom relief. As post‐viral neurological sequelae become more recognized, clinicians must consider cranial nerve involvement even in the absence of confirmatory testing or imaging abnormalities. A multidisciplinary approach remains essential for accurate diagnosis and effective management.

## 1. Introduction

Paresthesia localized to the infraorbital nerve distribution is a rare but clinically significant symptom, often resulting from trauma, dental pathology, maxillary sinusitis, or neurological conditions such as trigeminal neuralgia or postherpetic neuropathy [1, 2]. Less frequently, systemic, inflammatory, or immune‐mediated neuropathies may present with isolated trigeminal sensory involvement. Among emerging etiologies, post‐viral neuropathy, particularly following viral respiratory infections like COVID‐19, has gained recognition as a potential cause of cranial nerve disturbances, including isolated mononeuropathies of the trigeminal system [3]. This case illustrates the diagnostic challenge when presented with a case of acute paresthesia and underscores the importance of a multidisciplinary approach in the evaluation and management of atypical orofacial sensory disturbances.

The infraorbital nerve, a terminal branch of the maxillary division (V2) of the trigeminal nerve (cranial nerve V), supplies sensory innervation to the lower eyelid, upper lip, and midface [4]. Paresthesia in this distribution may manifest as anesthesia (total loss of sensation), dysesthesia (unpleasant or abnormal sensation), or allodynia (pain in response to a normally nonnoxious stimulus), either in isolation or in combination, and may occur alongside other cranial nerve deficits [5]. Given the anatomical course of the infraorbital nerve through the infraorbital canal and its close relationship to the maxillary sinus and dentoalveolar structures, pathology affecting adjacent dental, sinus, or soft tissue structures must be carefully excluded.

The differential diagnosis of infraorbital neuropathy is broad and requires a structured approach. Odontogenic causes include periapical infection, dental trauma, or complications from endodontic or surgical procedures. Traumatic etiologies may result from facial fractures or blunt force injury. Iatrogenic injury can occur following maxillary osteotomies, sinus surgery, cosmetic filler injections, or dental anesthesia. Neoplastic causes include benign or malignant tumors of the maxilla, sinus, or orbit causing compressive neuropathy. Sinus‐related inflammation, mucoceles, or chronic sinusitis may similarly affect the nerve. Systemic considerations include vasculitic neuropathies, demyelinating disease, metabolic disorders, and postinfectious or immune‐mediated neuropathies.

Recent literature has described an increasing number of post‐COVID‐19 neuropathic complications, including trigeminal neuralgia, small‐fiber neuropathy, and cranial mononeuritis. In a 2023 meta‐analysis, ~8.4% of patients with COVID‐19 reported trigeminal neuralgia, most commonly involving the ophthalmic and maxillary branches, with proposed mechanisms including direct viral invasion and postinfectious inflammation [6]. Peripheral neuropathy and myopathy were present in 56.3% of post‐COVID‐19 patients, with higher prevalence among those with symptoms, elevated CPK, and histories of hospitalization or prolonged respiratory illness [7]. SARS‐CoV‐2 is known to affect the nervous system via ACE2 receptor binding, cytokine release, microvascular injury, and possible reactivation of latent viruses [8].

However, in the absence of confirmatory SARS‐CoV‐2 testing at symptom onset and without a comprehensive neurological evaluation including electrodiagnostic studies or advanced neuroimaging, attribution of the neuropathy in this case to a post‐viral etiology remains presumptive rather than definitive. This distinction is critical, as isolated infraorbital neuropathy following COVID‐19 infection is rare and underreported, and alternative structural, infectious, and iatrogenic causes must be rigorously excluded prior to establishing causality.

Isolated infraorbital neuropathy following COVID‐19 remains rare and underreported. The complexity of diagnosing such presentations necessitates a multidisciplinary workup to exclude structural, infectious, or iatrogenic causes. Evaluation often necessitates a multidisciplinary approach involving dentistry, otolaryngology, neurology, radiology, and, when appropriate, infectious disease specialists. This case highlights the diagnostic complexity of isolated infraorbital neuropathy and supports inclusion of post‐viral neuritis within the differential diagnosis for unexplained orofacial sensory disturbances, while acknowledging inherent diagnostic limitations.

## 2. Case Presentation

A 34‐year‐old Caucasian male presented to the dental office with persistent unilateral paresthesia localized to the upper left lip and midface. His past medical history was significant for well‐controlled hypertension managed with lisinopril. He denied a history of diabetes mellitus, impaired glucose tolerance, peripheral neuropathy, autoimmune disease, vitamin deficiency, or prior neurologic disorders. He reported no tobacco or recreational drug use and only occasional alcohol consumption. There was no family history of neuropathic or demyelinating conditions.

**Figure 1:**
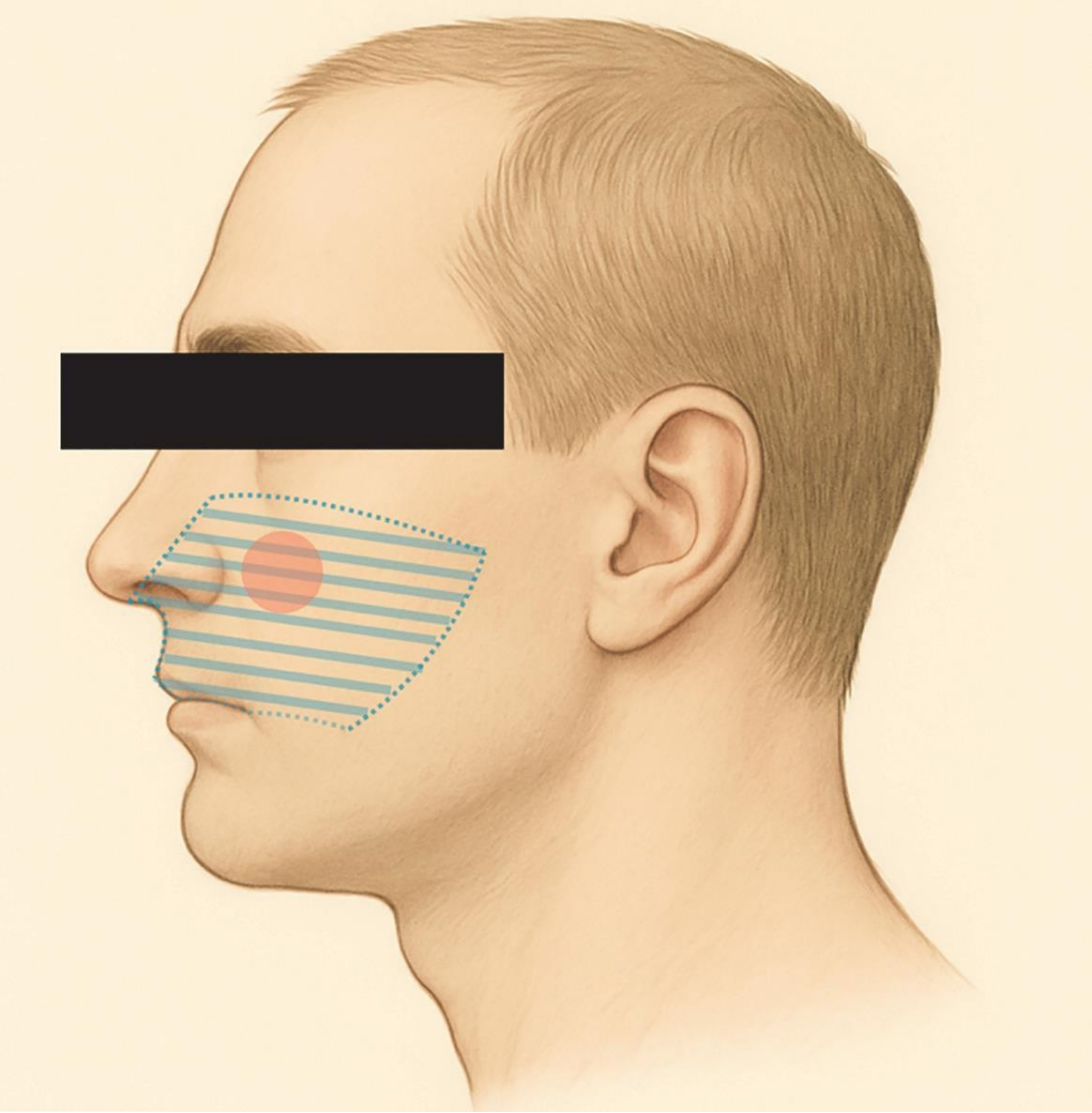
Extraoral–infraorbital nerve mapping. The dashed area represents the region of dysesthesia. The red shaded area represents the region of allodynia.

**Figure 2:**
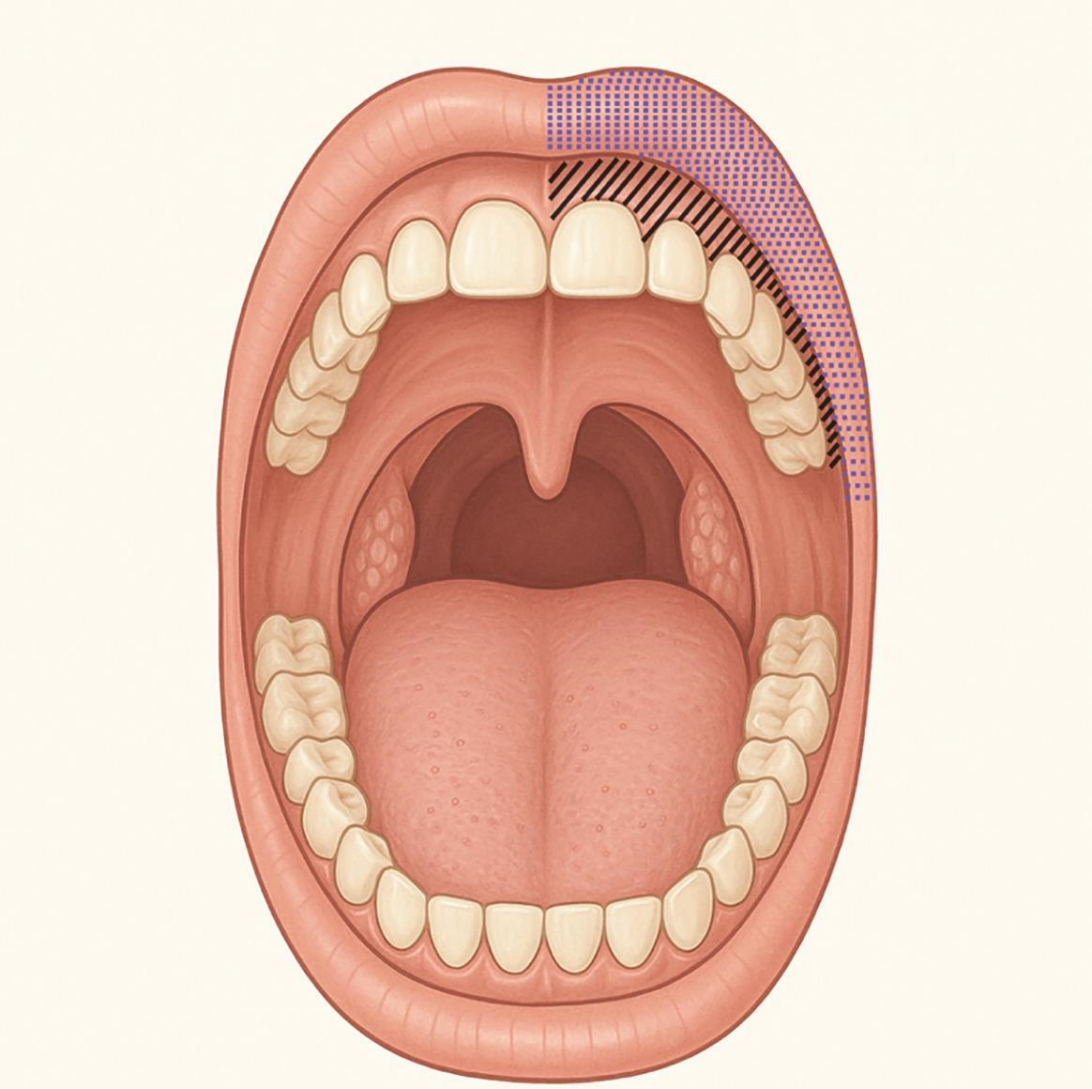
Intraoral–infraorbital nerve mapping. The polka dot area represents the region of dysthesia. The dashed area represents the region of allodynia.

The patient also reported focal allodynia with dull aching pain elicited by palpation over the left infraorbital foramen and dysesthesia of the left buccal maxillary attached mucosa, radiating mildly toward the zygomatic arch and left ear (Figures 1 and 2). He denied recent dental procedures, trauma, or known facial injury. The most common causes of infraorbital neuropathy, such as trauma, dental infection, sinus disease, and certain neurological conditions, were initially considered [9].

The patient described a persistent, unpleasant tingling and “raw” sensation that developed acutely on May 7, 2025, 1 week after onset of an upper respiratory illness that affected several members of his household (Table 1). He denied fever, anosmia, or cough but reported nasal congestion and fatigue. COVID‐19 infection was presumed based on symptom timing and epidemiologic exposure; however, no laboratory confirmation, including polymerase chain reaction or antigen testing, was obtained. As a result, attribution of the neuropathy to SARS‐CoV‐2 remains hypothetical and represents a key limitation of this case.

**Table 1:** Sequence of events.

Date	Event
April 26, 2025	Family members develop upper respiratory infection (suspected COVID‐19).
May 3, 2025	The patient develops upper respiratory symptoms.
May 7, 2025	Onset of paresthesia.
May 22, 2025	Emergency department visit: CT neck with IV contrast shows mild sinusitis; dexamethasone administered; referred to ENT.
June 5, 2025	ENT visit: Diagnosed with sinusitis; prescribed Augmentin and Medrol dose pack.
June 9, 2025	ENT second opinion: Nasal endoscopy performed; no abnormalities detected.
July 10, 2025	Dental evaluation: All teeth tested with cold and percussion tests; results normal; no dental pathology found.
July 21, 2025	Follow‐up dental visit: Infraorbital nerve block with 0.5% Marcaine and 1:200k epinephrine performed; anesthesia confirmed; paresthesia reproduced and pain relieved temporarily.
January 12, 2026	About 6‐month follow‐up: 90% improvement in symptoms with occasional mild flare ups and no new deficits.

He described the facial symptoms as a constant, unpleasant tingling and burning or raw sensation, rated as 4–5 out of 10 in intensity. Symptoms were continuous rather than episodic, with intermittent exacerbation upon touch or pressure to the infraorbital region. No specific relieving factors were identified, and symptoms were not worsened by mastication, temperature changes, or jaw movement. Symptoms had persisted for 2 months prior to his dental visit, in which time he was evaluated by other medical specialists.

On May 22, 2025, the patient presented to the emergency department, in which a CT neck with IV contrast revealed bilateral maxillary sinusitis, mild lingual tonsil hypertrophy, and a borderline reactive right cervical lymph node (Table 1). The patient was administered 10 mg dexamethasone intramuscularly and referred for outpatient otolaryngology follow‐up.

On June 5, 2025, the patient was prescribed a 10‐day course of amoxicillin‐clavulanate and a Medrol dose pack by the otolaryngologist to treat a working diagnosis of bilateral maxillary sinusitis (Table 1). Although these interventions led to improvement in sinus‐related symptoms, they had no effect on the facial paresthesia or allodynia.

He was then evaluated by a different otolaryngologist for a second opinion on June 9, 2025 (Table 1). Nasal endoscopy demonstrated no obstructive pathology or sinus drainage abnormalities. Oral examination and cranial nerve testing were grossly normal.

Although initial computed tomography imaging of the neck with intravenous contrast demonstrated bilateral maxillary sinusitis, subsequent otolaryngologic evaluation, including nasal endoscopy, revealed no evidence of active sinonasal inflammation, mucosal edema, purulence, or obstructive pathology. Additionally, directed medical therapy resulted in improvement of sinonasal symptoms without any corresponding improvement in facial paresthesia. Collectively, these findings argue against sinusitis as the primary contributor to the patient’s neuropathic symptoms. Magnetic resonance imaging of the trigeminal nerve or skull base was not obtained, which limits evaluation for subtle neural inflammation, perineural spread, or proximal trigeminal pathology and constitutes an additional diagnostic limitation.

**Figure 3:**
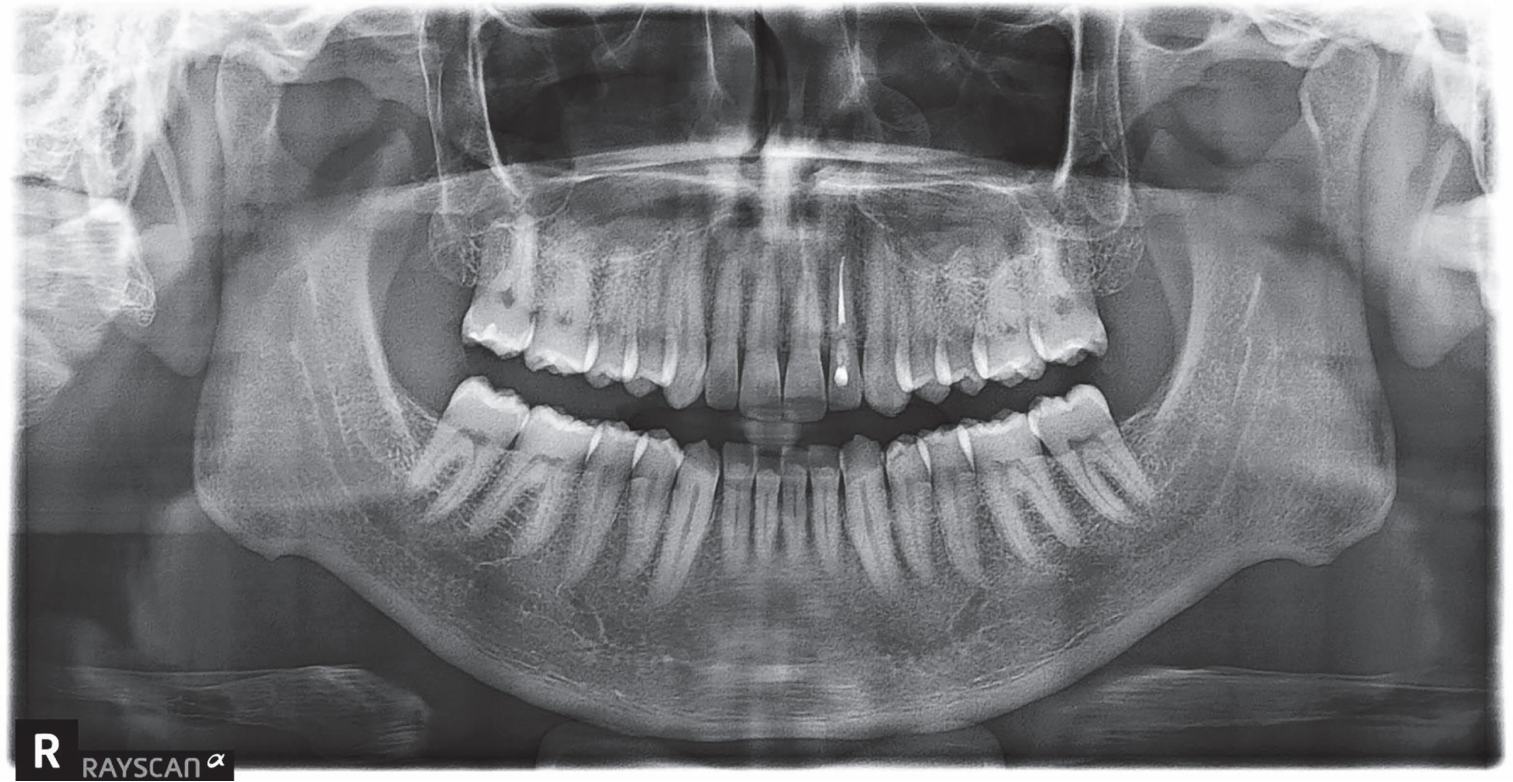
Panoramic radiograph.

**Figure 4:**
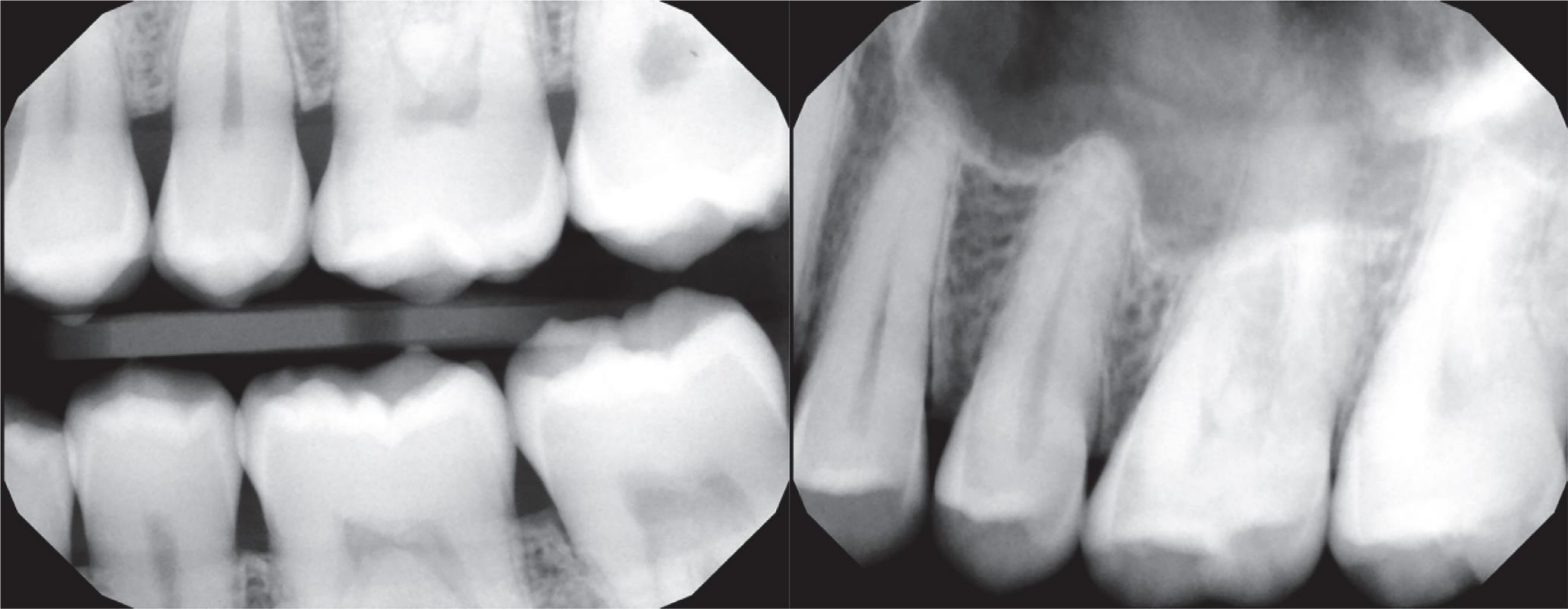
Upper left bitewing + periapical radiographs.

**Figure 5:**
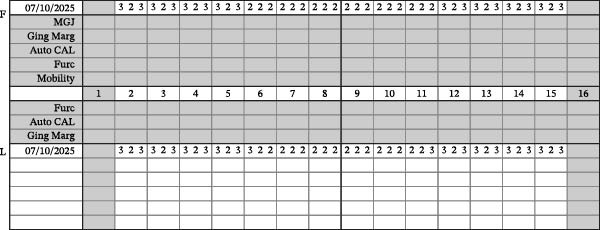
Periodontal chart.

**Figure 6:**
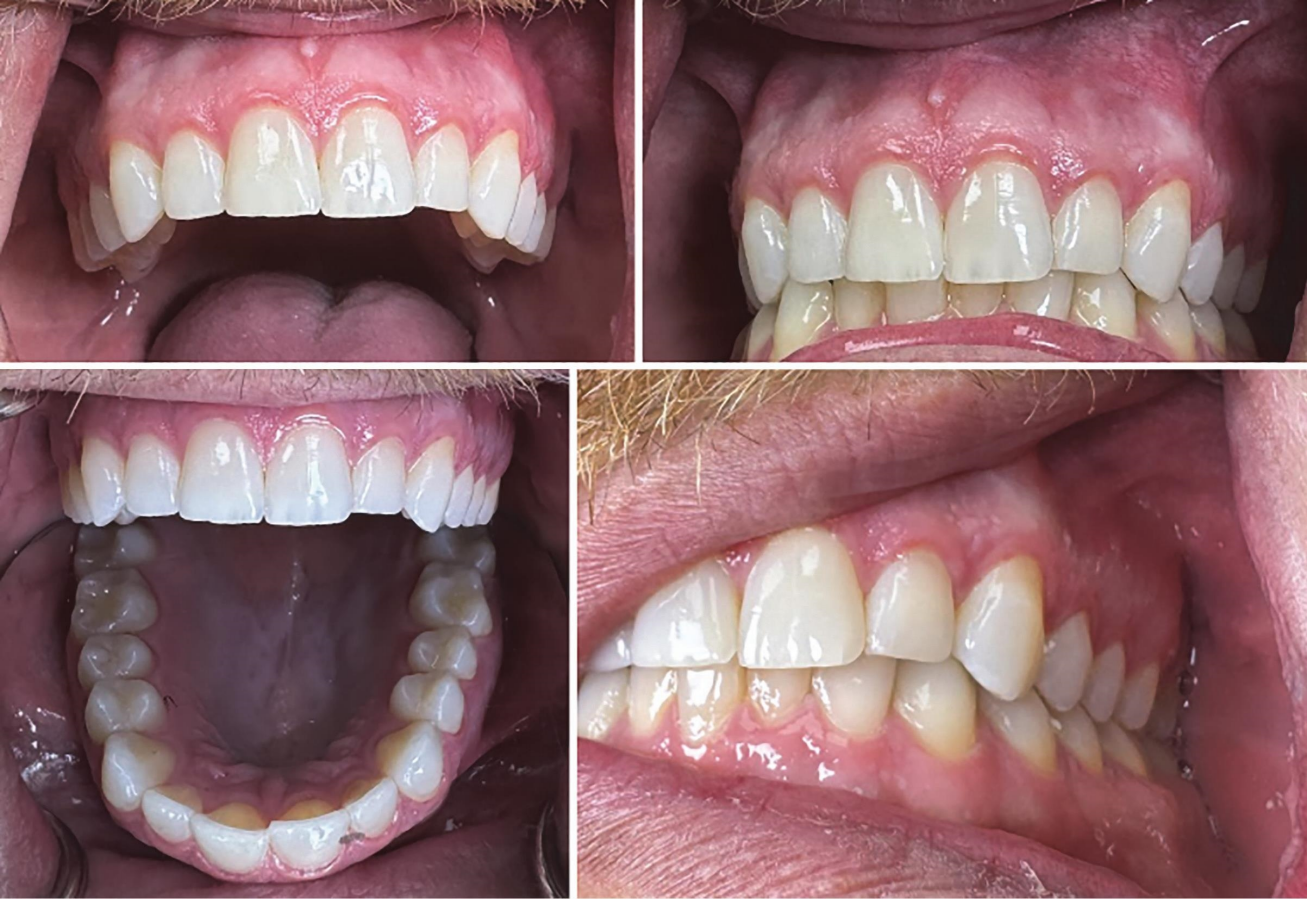
Clinical photos.

Finally, a comprehensive dental evaluation was performed, including intraoral photography, full‐mouth series radiographs, cone‐beam computed tomography (CBCT) imaging, and a detailed intraoral examination with endodontic testing and periodontal probing of all maxillary teeth in the affected region (Table 1). All findings were within normal limits, effectively excluding pulpal, periodontal, or osseous contributors to the sensory disturbance (Figures 3–6).

To confirm the affected nerve and assess therapeutic potential, an infraorbital nerve block was performed on July 21, 2025, using 1.7  mL of 0.5% bupivacaine with 1:200,000 epinephrine (Table 1). Prior to anesthesia, all maxillary teeth were again tested using cold and percussion, and results remained within normal limits. After administration, pulpal testing confirmed profound anesthesia in the anterior and middle superior alveolar (ASA/MSA) nerve distributions, indicating successful blockade of the infraorbital nerve and its branches [10].

During administration of the infraorbital nerve block, the patient reported transient reproduction of the original “raw” sensation at the injection site. This response was interpreted as a Tinel‐like phenomenon, suggesting localized irritation of the infraorbital nerve. Following injection, there was complete resolution of allodynia and dysesthesia, which persisted for ~6 h, consistent with the expected duration of action of 0.5% bupivacaine. Symptoms gradually returned as the anesthetic effect waned, supporting a peripheral source localized to the infraorbital nerve.

Importantly, the allodynia and dysesthetic symptoms resolved completely during the anesthetic window, confirming involvement of the infraorbital nerve and/or terminal branches of V2. Additionally, the patient reported a reproduction of the original “raw” sensation during the anesthetic injection, further localizing the nerve irritation.

Per these findings, the sensory disturbance was deemed to be confined to the distribution of the infraorbital nerve (a terminal branch of the maxillary division of the trigeminal nerve, V2). Given the absence of odontogenic, traumatic, or neoplastic pathology and the temporal correlation with a presumed viral infection, a diagnosis of post‐viral infraorbital paresthesia was favored. Nevertheless, this diagnosis remains one of exclusion, limited by the lack of virologic confirmation, incomplete neurologic evaluation, and absence of advanced neuroimaging.

Management was conservative and included reassurance, over‐the‐counter analgesics as needed, and clinical observation. Vitamin B‐complex supplementation was recommended, particularly vitamins B1, B6, and B12, which are known to support peripheral nerve healing. Vitamin B‐complex supplementation, particularly formulations containing thiamine, pyridoxal‐5‐phosphate, folate, and methylcobalamin, may ameliorate post‐viral neuropathies through synergistic effects on myelin synthesis, axonal regeneration, neurotransmitter homeostasis, mitochondrial energy metabolism, and modulation of neuroinflammatory and homocysteine‐mediated neurotoxic pathways [11, 12]. While the evidence for efficacy in post‐viral neuropathy is largely extrapolated from studies in diabetic and nutritional neuropathies, these vitamins are considered low risk and may facilitate symptom improvement over weeks to months. Typical recovery timelines in peripheral neuropathies vary widely but supplementation is often continued for several months to optimize nerve healing.

A neurology referral was also provided; however, a formal neurologic evaluation has not been completed. This introduces diagnostic uncertainty, as alternative neurologic etiologies, including small fiber neuropathy, immune mediated neuropathies, or early demyelinating disorders, cannot be definitively excluded in the absence of specialized neurologic assessment and ancillary testing.

Neuropathic pain medications such as gabapentin, pregabalin, and duloxetine were not initiated in this case, as symptom intensity was relatively mild, and pain was nondebilitating. The patient preferred to avoid potential adverse effects associated with these agents; additionally, initiation and management of such medications fall outside the typical scope of dental practice, leading to an initial conservative approach with observation given the stable and nonprogressive nature of symptoms.

No formal desensitization therapy or physical modalities were proposed at this time. Although desensitization and graded sensory retraining are commonly employed in neuropathic pain syndromes to modulate aberrant nerve signaling and improve function, such interventions were deferred pending neurologic evaluation and reassessment of symptom progression.

At evaluations over a 2‐month period following onset of symptoms, the patient’s paresthesia and allodynia remained stable and nonprogressive. No interval worsening of sensory disturbance was reported, and no improvement beyond transient relief following infraorbital nerve block was observed. Importantly, there was no development of motor deficits, expansion of sensory involvement beyond the infraorbital nerve distribution, emergence of additional cranial nerve abnormalities, or systemic neurologic symptoms during this period.

At the 6‐month follow‐up, the patient reported ~90% resolution of symptoms (Table 1). He described intermittent flare‐ups lasting several days, which were significantly milder than the initial presentation and did not interfere with daily activities. No progression of sensory disturbance or development of new neurologic symptoms was noted. The patient had not required additional medical or dental appointments since the prior evaluation. There were no changes in medical history, medications, or systemic health status.

Given the stability of symptoms and absence of red‐flag features, continued conservative management with clinical observation was elected. Further follow‐up is anticipated at regular intervals to monitor symptom trajectory. Additional diagnostic evaluation, including referral to neurology and advanced neuroimaging such as magnetic resonance imaging of the trigeminal nerve and skull base, will be pursued if any of the following occur: progression or worsening of sensory symptoms, development of motor weakness or additional cranial nerve involvement, transition to severe or functionally limiting neuropathic pain, or persistence of symptoms without improvement over an extended period.

This structured follow‐up strategy balances the low likelihood of acute structural pathology with the recognized potential for delayed or evolving neurologic manifestations in post‐viral cranial neuropathies, while maintaining readiness for escalation of care should clinical status change.

## 3. Discussion

Post‐viral cranial neuropathies, including trigeminal mononeuropathies, have increasingly been associated with SARS‐CoV‐2 infection. However, the overall incidence remains low, and causal relationships are often inferred rather than definitively established. A systematic review found that cranial nerve involvement in COVID‐19 patients was uncommon but notable, with facial and abducent nerves being the most frequently affected [13].

The trigeminal nerve, particularly its maxillary and mandibular branches, has been implicated in isolated cases of neuropathy following COVID‐19 infection. These cases often present with sensory disturbances such as numbness or pain in the affected areas. The exact mechanisms remain under investigation but potential factors include direct viral invasion, immune‐mediated inflammation, or post‐viral recovery processes. While the majority of patients experience transient symptoms, some report prolonged or recurring issues, highlighting the need for ongoing monitoring and research into the long‐term neurological effects of COVID‐19.

The persistent paresthesia localized to the distribution of the infraorbital nerve, in the absence of odontogenic, traumatic, or structural pathology, raises concern for post‐viral neuropathy or idiopathic infraorbital neuralgia. COVID‐19 has been implicated in various cranial neuropathic presentations, although in this case, a definitive diagnosis was not established [14].

The infraorbital nerve branch of V2 can be affected by trauma, sinus disease, malignancy, or surgical insult [5]. When these are excluded, post‐viral neuropathies should be considered, especially following upper respiratory infections. This case underscores the importance of recognizing isolated cranial mononeuropathies as possible, yet underreported, causes of infraorbital paresthesia.

Emerging reports suggest that SARS‐CoV‐2 may affect the nervous system through several mechanisms, potentially leading to a range of neurological complications, including sensory disturbances and cranial nerve involvement [15]. While the exact pathways remain under investigation, such presentations are increasingly recognized in both acute infection and post‐viral recovery. Clinicians should maintain a broad differential diagnosis when evaluating unexplained neuropathic symptoms in the context of recent viral illness.

A recent case report described infraorbital nerve neuropraxia following COVID‐19 infection, presenting as unilateral upper lip numbness and pain in the distribution of the maxillary branch of the trigeminal nerve (V2) [16]. This case shows that even mild COVID‐19 illness can lead to nerve‐related symptoms in the face. The numbness and discomfort were traced to the infraorbital nerve, which runs just below the eye and supplies sensation to the upper lip and cheek. These symptoms may be caused by lingering inflammation or irritation of the nerve after the infection and can easily be mistaken for dental or sinus problems.

In the present case, the working diagnosis of suspected post‐viral infraorbital neuropathy was supported by several clinical features, while acknowledging diagnostic limitations. Odontogenic causes were excluded based on the absence of dental pathology, normal pulp vitality testing, and lack of response to dental interventions. Sinus‐related etiologies were considered unlikely due to unremarkable sinus evaluation and the absence of sinonasal symptoms or radiographic abnormalities. There was no history of facial trauma, maxillofacial surgery, or local anesthetic injury that might account for iatrogenic or traumatic nerve insult. Systemic neuropathic conditions were not clinically suggested, as symptoms were focal, unilateral, and isolated to the infraorbital nerve distribution without evidence of polyneuropathy or systemic neurologic disease. Nonetheless, advanced neuroimaging and formal neurologic consultation were not performed, precluding definitive exclusion of all alternative etiologies.

Compared with previously reported cases of post‐COVID‐19 trigeminal neuropathy, this presentation demonstrates both overlap and distinction. Published reports of trigeminal V2 involvement following SARS‐CoV‐2 infection typically describe sensory disturbances such as facial numbness, dysesthesia, or neuropathic pain, often occurring weeks after mild respiratory illness [14, 16]. Some cases involve multiple trigeminal branches or coexist with other cranial nerve deficits, whereas this patient exhibited isolated infraorbital nerve paresthesia without additional cranial nerve involvement, dental pathology, or structural abnormalities. The persistent yet nonprogressive nature of symptoms further differentiates this case from more acute inflammatory neuropathies reported in the literature. These features suggest that localized trigeminal mononeuropathies may represent an underrecognized post‐viral manifestation.

Prognosis in post‐viral neuropathies is variable. While many cases demonstrate gradual improvement or spontaneous resolution over weeks to months, persistent symptoms lasting months or even years have been documented, particularly in cranial mononeuropathies [13, 16]. Accordingly, long‐term follow‐up is warranted, especially if symptoms progress or fail to improve. This case underscores that although recovery is common, chronic sensory disturbances remain possible.

Management of post‐viral neuralgia is largely symptomatic. In this case, over‐the‐counter analgesics, observation, and adjunctive vitamin B‐complex therapy were selected due to stable symptoms and absence of concerning features. B‐complex supplementation, particularly vitamins B1, B6, and B12, supports nerve metabolism, remyelination, and axonal repair and may aid recovery, although evidence specific to post‐viral cranial neuropathy remains limited [11, 12].

Emerging therapeutic approaches for nerve repair include low‐level laser therapy (LLLT), which may stimulate cellular regeneration and modulate inflammation, and pulsed electromagnetic field (PEMF) therapy, shown to enhance nerve healing in experimental models [17, 18]. In refractory cases of cranial neuropathy, peripheral nerve blocks using corticosteroids or long‐acting local anesthetics such as bupivacaine have provided symptom relief and diagnostic value [10, 19]. Notably, exosome‐based treatments and regenerative biologics are being investigated for their potential to promote axonal repair and reduce neuroinflammation in chronic neuropathic conditions [20, 21]. However, due to limited access and relatively mild symptom severity, these modalities were not pursued in this case.

Overall, this case highlights the diagnostic challenges of persistent infraorbital paresthesia following viral illness and emphasizes cautious diagnostic framing, systematic exclusion of common etiologies, and interdisciplinary collaboration. Isolated infraorbital neuropathy should be considered in the differential diagnosis of unexplained midfacial sensory disturbances, while recognizing that definitive causation may remain elusive.

## 4. Conclusion

This case exemplifies the diagnostic challenges of persistent facial paresthesia following presumed viral illness. Infraorbital nerve involvement, though rare, should be included in the differential even in the absence of confirmatory testing or imaging. Continued interdisciplinary collaboration and clinical awareness remain crucial. As long as COVID‐19 continues to evolve, clinicians must remain vigilant for localized cranial nerve dysfunction such as infraorbital neuritis. A collaborative multidisciplinary approach involving dental, ENT, and neurology specialists can aid accurate diagnosis and tailored management [22]. In particular, neurological evaluation and additional imaging such as magnetic resonance imaging are important to help exclude alternative etiologies, including demyelinating, autoimmune, or neoplastic processes. Further research is needed to explore the pathophysiology, prevalence, and treatment of post‐COVID‐19 cranial neuropathies.

## Author Contributions

All authors contributed to the initial drafting of the manuscript. Nam Nguyen conceived the project in the role of clinical provider and was responsible for data acquisition, clinical communication, fabrication of tables and figures, and identification of relevant scientific sources. Willow Meline contributed to data acquisition, formatting and organization, technical content, and performed the final review and editing of the manuscript. Elborz Safarzadeh provided overall project oversight, guided the scientific methodology for data acquisition, managed communications with the publisher, and manuscript formatting.

## Funding

No funding was received for this manuscript.

## Disclosure

All authors reviewed and approved the final manuscript.

## Consent

Written informed consent was obtained from the patient to publish this case report per the journal’s patient consent policy.

## Conflicts of Interest

The authors declare no conflicts of interest.

## Data Availability

The data that support the findings of this study are available upon request from the corresponding author. The data are not publicly available due to privacy or ethical restrictions.
